# A large panel of chicken cells are invaded *in vivo* by *Salmonella* Typhimurium even when depleted of all known invasion factors

**DOI:** 10.1098/rsob.210117

**Published:** 2021-11-17

**Authors:** S. M. Roche, S. Holbert, Y. Le Vern, C. Rossignol, A. Rossignol, P. Velge, I. Virlogeux-Payant

**Affiliations:** INRAE, Université de Tours, ISP, 37380 Nouzilly, France

**Keywords:** *Salmonella*, poultry, host–pathogen interaction, invasion, gall bladder

## Abstract

Poultry are the main source of human infection by *Salmonella*. As infected poultry are asymptomatic, identifying infected poultry farms is difficult, thus controlling animal infections is of primary importance. As cell tropism is known to govern disease, our aim was therefore to identify infected host–cell types in the organs of chicks known to be involved in *Salmonella* infection and investigate the role of the three known invasion factors in this process (T3SS-1, Rck and PagN). Chicks were inoculated with wild-type or isogenic fluorescent *Salmonella* Typhimurium mutants via the intracoelomic route. Our results show that liver, spleen, gall bladder and aortic vessels could be foci of infection, and that phagocytic and non-phagocytic cells, including immune, epithelial and endothelial cells, are invaded *in vivo* in each organ. Moreover, a mutant defective for the T3SS-1, Rck and PagN remained able to colonize organs like the wild-type strain and invaded non-phagocytic cells in each organ studied. As the infection of the gall bladder had not previously been described in chicks, invasion of gall bladder cells was confirmed by immunohistochemistry and infection was shown to last several weeks after inoculation. Altogether, for the first time these findings provide insights into cell tropism of *Salmonella* in relevant organs involved in *Salmonella* infection in chicks and also demonstrate that the known invasion factors are not required for entry into these cell types.

## Graphical abstract

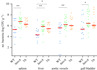

## 1. Background

*Salmonella* spp. are among the most important foodborne pathogens. From a public health perspective, according to the World Health Organization, *Salmonella* spp. are among the 31 diarrhoeal and/or invasive agents (viruses, bacteria, protozoa, helminths and chemicals) displaying the highest capability of triggering intestinal or systemic diseases in humans. Most cases of salmonellosis are mild, but sometimes the disease is life-threatening and salmonellosis is the third leading cause of death among food-transmitted diseases [1].

The two most commonly reported non-typhoïdal serovars *Salmonella enterica* subsp. *enterica* serovar Enteritidis and *Salmonella enterica* subsp. *enterica* serovar Typhimurium (including its monophasic variant) account for almost 80% of human cases occurring in the EU [2]. Depending on host factors and serovars, *Salmonella* can induce a wide range of diseases ranging from systemic to asymptomatic infections and gastroenteritis [3]. In humans, localized infections can be followed by bacteraemia in 3–10% of cases [4].

Animals are the primary source of these pathogens and humans become infected mainly by ingesting contaminated food. Poultry meat and eggs are the main source of human *Salmonella* contamination. In 2010, Knight-Jones *et al*. [5] reported that poultry was implicated as the source of outbreak in 10.4% of the total cases worldwide. Since 2018, *Salmonella*-positive single samples have represented the highest prevalence following official control investigations [2]. The detection and eradication of *Salmonella* in poultry is difficult because *Salmonella* infection mostly induces an asymptomatic infection, accompanied by high faecal excretion, which is a source of transmission [6]. Consequently, contaminated poultry flocks have to be eradicated and all derived products destroyed, resulting in high economic losses. It is therefore particularly important to control animal infection not only to avoid economic consequences but also to prevent the negative impacts on human health.

To establish infection in their hosts, *Salmonella* have to interact with several phagocytic and non-phagocytic eukaryotic cells. Invasion of these cells is considered as one of the key steps in *Salmonella* pathogenesis. The best-known invasion process requires the type III secretion system-1 (T3SS-1) encoded by *Salmonella* pathogenicity island 1 (SPI-1). The T3SS-1 is a needle-like structure, which injects bacterial effector proteins directly into the host cytosol to manipulate the cell cytoskeleton, allowing bacterial internalization into non-phagocytic cells [7]. Two other *in vitro* entry pathways, involving the Rck and PagN invasins, have also been described in *Salmonella* [8–10]. Contrary to the T3SS-1, each invasin interacts with a eukaryotic receptor, EGFR and the heparinated proteoglycan for Rck and PagN, respectively [11,12]. *In vivo*, several studies particularly in mice have reported the key role of the T3SS-1 for *Salmonella* to cross the intestinal barrier [13,14]. Nevertheless, infections in the absence of T3SS-1 in mice, chicks and calves have also been described in several papers in which a mutant defective for the T3SS-1 was shown to colonize its host as efficiently as its wild-type parent [15–20]. This has also been observed in humans in whom food-borne disease outbreaks have been described with *Salmonella* Senftenberg isolates which lack segments of SPI-1 [21]. Similarly, a study performed by Suez *et al.* [22] comparing the pathogenicity of different non-typhoïdal strains concluded that *Salmonella* virulence factors, including multiple T3SS effectors, were absent from several bacteraemia isolates suggesting they are not required for invasive infection. Less is known about the role of the Rck and PagN invasins *in vivo*, but *pag*N (formerly *iviVI-A*) and *rck* mutants are both less competitive than their wild-type parent in mice [23–25]. However, apart from these roles identified at the organ level, very little is known about the cells targeted by these invasion factors and this is particularly the case in farm animals.

This topic is crucial because cell and tissue tropism governs disease in many models [26,27]. Moreover, some studies have shown that depending on the entry mechanism both bacterial behaviour and host response are different [28]. It is therefore important to identify the host cells targeted by *Salmonella* and the different entry routes used by this pathogen to invade the different host cells. In order to improve understanding of how *Salmonella* infect chicks, our aim was to identify the cells in four different organs known to be involved in *Salmonella* infection of this species that could be targeted *in vivo* by this pathogen when expressing or not the known invasion factors. For this purpose, we used a fluorescent *S.* Typhimurium wild-type strain and its fluorescent mutant derivatives deleted of either the T3SS-1 alone or of the three known invasion factors (T3SS-1, Rck and PagN) to infect chicks in the coelomic cavity. The use of the mutants aimed to investigate the impact of these factors on the invasion of the cell types infected by *Salmonella*. Identification of phagocytic and non-phagocytic cells and their invasion by the different bacteria were monitored using flow cytometric analyses and confocal microscopy.

## 2. Methods

### 2.1. pFPV-TurboFP650 plasmid construction

Gene encoding TurboFP650 was amplified from the plasmid pTurboFP650-N (Evrogen, Moscow, Russia) with primers TurboFP650-XbaI 5′-TGCTCTTAGATTTAAGAAGGAGATATAGATATGGGAGAGGATAGCGAGCTG-3′ and TurboFP650-SphI 5′-CATGCATGCTTAGCTGTGCCCCAGTTTGCTAGG-3′. Then, the PCR product and the pFPV25.1 plasmid [29] were restricted by XbaI and SphI restriction enzymes, ligated and transformed into *Escherichia coli* MC1061 [30]*.* pFPV-TurboFP650 recombinant plasmids were selected on Trypticase Soya Agar (TSA—BD Difco, Franklin Lakes, USA) containing 100 µg ml^−1^ of carbenicillin (Sigma-Aldrich, Saint-Quentin Fallavier, France) and clones which showed a purple colour were selected for restriction analysis. Clones with good restriction profiles were then sequenced to confirm the absence of mutations in the TurboFP650 coding sequence.

### 2.2. Strains used and inocula preparation

The pFPV-TurboFP650 plasmid was introduced in *S.* Typhimurium 14028 wild-type (WT), the Δ*invA*::*kan* mutant (Δ*invA*; T3SS-1 defective) or the Δ*invA::kan* Δ*pagN::cm* Δ*rck* mutant (3Δ) [31].

To prepare the inocula, the strains were cultured in Trypticase Soya Broth (TSB—BioMérieux) supplemented with carbenicillin 100 µg ml^−1^ for 24 h at 37°C with shaking. The cultures were centrifuged at 4500 *g* for 20 min at 20°C and the pellets were suspended in phosphate buffered saline (PBS) containing 50% glycerol. The bacterial suspensions were then aliquoted, frozen and stored at −80°C. The frozen aliquots from the same initial inoculum were used throughout the experiments.

### 2.3. Experimental infection

Five-day-old PA12 White Leghorn chicks, provided by the Experimental Platform for Infectious Disease (UE 1277—INRAE), were inoculated in the coelomic cavity with 0.2 ml of bacterial suspension. On the day of inoculation, a frozen aliquot of the inoculum was thawed. Bacteria concentrations were standardized turbidimetrically and diluted to a concentration of 6 × 10^7^ CFU per 0.2 ml in PBS. Chicks were maintained in medium isolator systems (0.83 m^2^) with controlled environmental conditions (feed, water, temperature, air humidity and lighting scheme) for 2 days before sacrifice by decapitation and bleeding. To follow the persistence in the gall bladder, the inoculation dose was 3 × 10^7^ CFU per chick, in order to reduce the mortality of chicks observed with the higher dose. The kinetics of organ colonization was monitored each week over a period of 36 days.

### 2.4. Enumeration of bacterial load in infected organs

On the day of sacrifice, control animals of the same age (i.e. not inoculated) were provided by the Experimental Platform for Infectious Disease. Spleens, livers, gall bladders and the aortic vessels were collected aseptically from each animal for quantification of bacterial load.

To determine the bacterial load, organs were homogenized in TSB and serial 10-fold dilutions were plated on TSA or *Salmonella*–*Shigella* medium supplemented with carbenicillin 100 µg ml^−1^. The colonies per plate were counted after incubation for 24 h at 37°C. Counts were expressed as log (CFU) per gram of organ.

### 2.5. Preparation of cells for flow cytometry

For the flow cytometric analyses, organ-specific samples were obtained by pooling the spleens, livers, aortic vessels and gall bladders of the different chicks in Hanks’s buffered saline solution (HBSS) without Ca^2+^ and Mg^2+^ in the dark at 4°C in order to be able to analyse at least 200 000 cells for each organ. Independent infections were repeated at least three times. Gall bladders and aortic vessels were cut into small pieces and samples put in collagenase A (0.3%—Sigma)—dispase I (1 U ml^−1^—Sigma)—HBSS for 30 min at 37°C. The whole purification process was performed at 4°C. All organs were then homogenized in HBSS using syringe plungers and filtered through 40 µm-mesh cell strainers (BD Falcon, Tewksbury, USA), before being transferred into a 50 ml centrifuge. After centrifugation at 1000 *g* for 15 min, cells were washed, resuspended in HBSS at approximatively 5.10^6^–1.10^7^ cells ml^−1^ and maintained in the dark at 4°C.

### 2.6. Flow cytometric analyses

Cells were characterized according to the antibodies available in poultry (electronic supplementary material, table S2). Mouse Anti-Chicken antibody, clone KUL01 specifically recognizes chicken monocytes, macrophages and interdigitating cells [32]. Anti-CT3 antibody targets the avian homologue of the CD3-antigen, a common antigen used to identify T lymphocytes [33]. Clone AV20 antibody recognizes the antigen Bu-1, a chicken B-cell marker, commonly used to identify B lymphocytes [34]. Mouse anti-chicken CD41/61 clone 11C3 recognizes chicken integrin CD41/61 that is expressed on chicken thrombocytes and cells of the thrombocyte lineage [35]. Mouse anti L-CAM antibody recognizes an 81 kDa N-terminal tryptic fragment of L-CAM, an epithelial cell marker, from embryonic chicken liver plasma membranes [36] and finally VE-cadherin is an intercellular junction marker of endothelial cells. This is a synthetic peptide corresponding to human VE-cadherin amino acids from position 750 to the C-terminus conjugated to keyhole limpet haemocyanin. Rabbit polyclonal antibody anti-VE-cadherin clone reacts with mouse, chicken and human VE-cadherin.

Anti-Bu-1 and the anti-CD3, that allow B and T lymphocytes to be identified, were FITC conjugated. Antibodies allowing monocytes–macrophages, thrombocytes, epithelial and endothelial cells to be identified, required Alexa Fluor 488 conjugated anti-secondary anti-mouse or anti-rabbit antibodies. The endothelial cell samples were pre-treated with 20% horse serum. The primary antibodies were incubated with cells for 90 min at 4°C in the dark and then rinsed in HBSS. When necessary, secondary antibodies were added for 90 min at 4°C in the dark, and then rinsed. Appropriate isotype-control antibodies (electronic supplementary material, table S2) were used to determine the levels of unspecific staining in all the experiments. Parallel samples were stained with a Fixable Viability Dye Cell Staining eFluor 450 (eBioscience, San Diego, USA) to determine the settings for a live cell gate based on light scatter properties. All samples were then filtered through 60 µm nylon Blutex immediately before flow cytometric analyses were performed using a BD LSR Fortessa X-20 (BD Biosciences, San Jose, CA, USA). BD FACSDiva software (v. 8.0.2, RRID:SCR_001456) was used to analyse the cytometric data. Infected and control samples were manipulated under the same conditions.

### 2.7. Identification and relative quantification of infected and non-infected cells by flow cytometry

For each sample, dot plots were analysed. The intensity of green fluorescence (FITC or Alexa Fluor 488) on the vertical axis was plotted against the intensity of red fluorescence (TurboFP650) on the horizontal axis. Labelled infected cells thus emitted both green and red fluorescence. They were revealed as dots in the upper right-hand part of the graph. For each experiment in each organ, a gate was determined removing inappropriate labelling—debris based on morphological criteria. Regions were set according to uninfected control samples and isotype-control staining. In order to have quantitative results, 200 000 events were analysed for each sample for all staining. Examples are provided for some cell-type/organ labelling: for monocytes–macrophages (electronic supplementary material, figure S1) and thrombocytes (electronic supplementary material, figure S2) in the gall bladder, B lymphocytes (electronic supplementary material, figure S3) and T lymphocytes (electronic supplementary material, figure S4) in the spleen, epithelial cells in the liver (electronic supplementary material, figure S5), and endothelial cells in the aortic vessels (electronic supplementary material, figure S6). Quantification of the percentage of positively labelled cells was then calculated by subtracting the number of cells in the control areas from those in the positive labelled areas. The positive labelling areas of B and T lymphocytes cells were established using a control mouse IgG1-FITC conjugate, whereas the positive labelling areas of monocytes–macrophages, thrombocytes and epithelial cells were determined with a control mouse IgG1-Alexa Fluor 488 conjugate. For the labelling of the endothelial cells, we used rabbit IgG, followed by a secondary antibody, an anti-IgG-Alexa Fluor 488 conjugate. The total percentages of infected cells which were positively labelled or unlabelled were also determined. All negative responses were scored at 0.001% to account for the threshold and to allow for a logarithmic representation of the results. The medians are represented by a red dash.

### 2.8. Purification of the infected cells and confocal laser-scanning analysis

Cells were sorted using a high-speed cell sorter, MoFlo Astrios^EQ^ (Beckman Coulter Inc, Brea, CA, USA) equipped with four lasers: violet (405 nm), blue (488 nm), yellow-green (561 nm) and red (640 nm) and placed under a class II biological safety cabinet. We used a 90 µm nozzle and selected a sheath pressure of 40 psi. Sorted cells were collected in 1.5 ml Eppendorf tubes containing 350 µl of HBSS medium supplemented with 10% fetal calf serum to limit cell stress.

After cell sorting, samples were deposited on glass coverslips and centrifuged with a cytospin at 200 r.p.m. for 10 min. Cells were then fixed in formaldehyde 4% for 10 min. Nucleus staining was performed with DAPI 1 µg ml^−1^ for 1 min and coverslips were mounted on slides with fluorescent mounting medium (Dako). Cells were observed under a SP8 confocal laser-scanning microscope equipped with an HCP PL APO 100×/1.44 Oil CORR CS immersion objective (Leica). Z-stacks were re-sliced horizontally and vertically to obtain the projections of perpendicular views from three-dimensional images, providing a view of all bacteria in the cells, using Las AF lite 2.6.3 build 8173 software (Leica application Suite X, RRID:SCR_013673).

### 2.9. Immuno-histochemistry (IHC)

Chick gall bladders were fixed in 4% buffered paraformaldehyde (Electron Microscopy Sciences, Hatfield, England) at 4°C for 24 h. They were then processed by routine methods, they were paraffin (Surgipaht Paraplast Plus Leica, Richmond, USA) embedded, cut in sections (thickness, 5 µm), and stained for IHC with HRP detection. All samples were incubated at room temperature. The primary antibody was an anti-*Salmonella* lipopolysaccharide marker: rabbit anti-*Salmonella* O:4,5 (1/100—Diagnostics Pasteur, Marne-la-Coquette, France). The tissue sections were dewaxed in Histosol (Shandon, Cergy Pontoise, France), rehydrated in a decreasing series of ethanol, rinsed and rehydrated in tap water. Sections were treated with heat-induced epitope retrieval, 10 mM sodium citrate buffer, pH 6, 121°C, for 15 min. The tissues were then rinsed in tap water. The endogen peroxidase was blocked in 1% hydrogen peroxide and methanol for 30 min. Preparations were rinsed in PBS with 1% skimmed milk and 0.05%Tween 20 (PBSTM), blocked in 20% goat serum −30% fetal calf serum—PBS for 20 min. They were then incubated with a primary antibody for 60 min and rinsed in PBSTM, followed by N-Histofine rabbit, HRP (Nichirei Biosciences, Tokyo, Japan) for 30 min. At the end, samples were rinsed in PBSTM, incubated with chromogen (diaminobenzidine, liquid DAB) (DAB Quanto, Lab Vision Corporation, Fremont, USA) for 5 min, counterstained with haematoxylin of Harris (Merck, KGaA, Darmstadt, Germany), rinsed in tap water, dehydrated in successive ethanol baths (50°, 70°, 90° and absolute, each for 2 min), cleared in histosol and mounted on coverslips with Eukitt (ORSAtec, Bobingen, Germany).

Tissues were examined and photographed with a light microscope Eclipse 80i, Nikon with DXM 1200C digital camera (Nikon Instruments, Europe, Amsterdam, The Netherlands) and NIS-Elements D Microscope Imaging Software (NIS-Elements, RRID:SCR_014329).

### 2.10. Statistical analyses

A Kruskal–Wallis test was conducted to examine the differences in the levels of organ colonization, followed by a Dunn's multiple comparisons test (GraphPad Prism v. 6.07 for Windows, GraphPad, www.graphpad.com, RRID:SCR_002798). Significance was *p < 0.05 and **p < 0.01.

For the flow cytometric analyses, asymptotic two-sample Fisher–Pitman permutation tests (one-way test) were performed with R software, package Rcmdr v. 2.5.3 (R Project for Statistical Computing, RRID:SCR_001905). Significance was *p < 0.05 (http://www.r-project.org, http://socserv.socsci.mcmaster.ca/jfox/Misc/Rcmdr/).

## 3. Results and discussion

### 3.1. Mutant strains defective for the T3SS-1 or the three known invasion factors inoculated by the intracoelomic route colonize chicks more effectively than their wild-type parent strain

**Figure 1.**
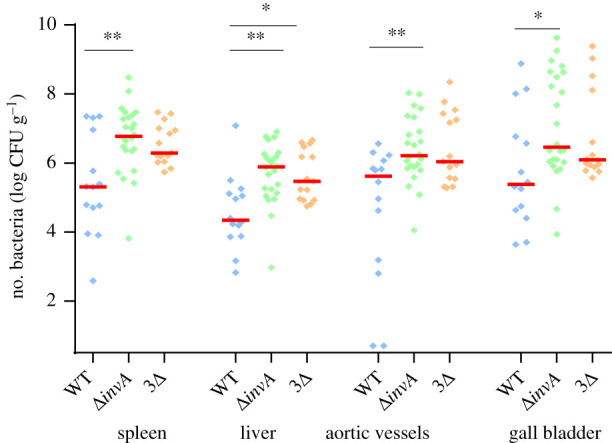
Level of different *S.* Typhimurium strains in organs of chicks after intracoelomic inoculation. Five-day-old chicks were inoculated in the coelomic cavity with around 6 × 10^7^ CFU per chick with *S*. Typhimurium 14028 turboFP650 wild-type strain (WT; blue diamond), Δ*invA*::*kan* mutant strain (Δ*invA*; T3SS-1 defective; green diamond) or the Δ*invA::kan* Δ*pagN::cm* Δ*rck* mutant strain (3Δ; T3SS-1, Rck, PagN defective; yellow diamond). Two days post infection, spleens, livers, aortic vessels and gall bladders were removed aseptically from each animal for quantification of bacterial load. Results are expressed as number of bacteria per gram of organ (log CFU per gram of organ). The medians are represented by a red dash. A Kruskal–Wallis test was conducted, followed by Dunn's multiple comparisons test (GraphPad Software). Significance was *p < 0.05 and **p < 0.01.

Previous work has shown that a Δ*invA* mutant strain (T3SS-1 defective strain) and a strain deleted for the three known invasion factors (3Δ) remained invasive for several eukaryotic cell lines compared to the wild-type strain [31]. To determine the ability of our wild-type mutant strains to invade several host cells *in vivo*, we infected chicks in the coelomic cavity (also abusively referred as intraperitoneal route) in order to have a sufficient number of *Salmonella* colonizing the targeted organs to allow infected cells to be identified in these organs. The first step was to evaluate the ability of these different strains to colonize vessels and different organs of chicks (i.e. the spleen, liver and gall bladder; figure 1). The vessels were chosen because *Salmonella* is able to spread through the body via the blood, and the spleen and liver as they are colonized by *Salmonella* in chicks [37,38]. Finally, we decided to study the gall bladder as well because Gonzalez-Excobedo & Gunn [39] demonstrated that the gallbladder epithelium contributed to establishing and maintaining chronic carriage in mice. To ensure that the levels of bacteria were not related to the presence of *Salmonella* in the blood, all chicks were bled.

The first observation is that all strains were able to infect all the organs and vessels. For some animals, the infection rate even reached 8 log CFU g^−1^, particularly for the gall bladder. Moreover, all organ colonization levels were higher after inoculation with the Δ*invA* strain than with the wild-type strain (29, 35, 3.9 and 12 times greater in the spleen, liver, aortic vessels and gall bladder, respectively). All these differences were statistically significant. While the 3Δ strain also colonized these organs and vessels more effectively than the wild-type strain (9.5, 13, 2.6 and 5.1 times greater in the spleen, liver, aortic vessels and gall bladder, respectively), a statistically significant difference was only identified in the liver. No significant statistical difference could be observed between the two mutants, beside the fact that the log CFU of bacteria g^−1^ for the 3Δ strain was always inferior to that of organs for the Δ*invA* strain. The levels of CFU recovered in the gall bladder and the aortic vessels should be highlighted, as they have rarely been studied in the past. These results demonstrate that all the organs and vessels tested were colonized by wild-type *S.* Typhimurium and that the two mutant strains (Δ*invA* or 3Δ) colonized these organs of chicks after intracoelomic inoculation at least at the same level as the wild-type strain.

This latter result could be attributed to the route of inoculation. Indeed, *Salmonella* injected into the coelomic cavity easily reach systemic sites such as the spleen and the liver. They could also reach the gall bladder through the vasculature or the hepatic duct [40]. *In vivo* studies have demonstrated that the T3SS-1 is primarily associated with the early stage of infection during which it translocates T3SS1 effectors across the host intestinal epithelial cell membrane and stimulates intestinal inflammation [13,14,41–43], thus playing an important role in *Salmonella* colonization after animals are inoculated orally. Our results show that, in chicken, the colonization of systemic organs can be independent of this type III secretion system as no difference in colonization between a T3SS-1 mutant and its wild-type parent was observed as described after intraperitoneal or intravenous inoculation of mice. In our case, one hypothesis that could explain the higher colonization of the mutant strains compared to the wild-type strain is that after intracoelomic inoculation the absence of the T3SS-1 could induce a lower immune system alert, in particular a lower inflammatory response and consequently less bacteria being killed. Indeed, SPI-1 genes are involved in the regulation of the host immune response, for example the host inflammatory response [44], immune cell recruitment [45] and apoptosis [46,47]. Moreover, we already know that a SPI-1 mutant and also a *phoP* mutant, not expressing PagN like our 3Δ mutant, did not stimulate an inflammatory response in chick caeca [48].

Cytometric analyses and microscopy were then performed in order to determine whether *Salmonella* was within the cells of the different organs and vessels and to identify the cell-types infected. The infectious dose of 6 × 10^7^ CFU per chick used for the previous *in vivo* experiment, represented a good compromise between the infectious dose and the period of slaughter (2 days), to enable enough intracellular bacteria to be detected for flow cytometry analyses. The animals were bled to reduce the number of red blood cells and allow better detection of organ cells. The concentration of *S*. Typhimurium-TurboFP650-wild-type (STM-Turbo FP650-WT) strain was checked in the blood of six animals. An average of 1.95 ± 1.09 log CFU ml^−1^ was found.

### 3.2. STM-Turbo FP650-WT and its mutant strains were within the cells and did not only adhere to the cells

As our aim was to identify cells infected by *Salmonella*, we first assessed whether our protocol allowed us to identify intracellular bacteria or not. Indeed, flow cytometry is useful for quantitative analyses but it does not enable bacteria to be localized as either adherent or intracellular. According to our protocol, it was highly unlikely that *Salmonella* would only be present extracellularly due to the methods used to purify and mechanically separate the cells, including filtrations and washings and, for some organs enzymatic cleavage with two different enzymes (collagenase and dispase) was also performed for cell purification. Theoretically, after all these treatments related to organ dissociation, only a few bacteria would remain adhered, suggesting that the large majority were intracellular. However, in order to confirm this, cell sorting based on the labelling of the cells and confocal analyses were carried out for each cell type of each organ. For flow cytometry, regions corresponding to infected cells were identified with the PE-cy5 canal and were set according to uninfected control samples. The Alexa fluor 488 canal was used to identify the cell types according to the isotype-control staining. One example for each cell type is given in electronic supplementary material, figures S1–S6. Double-labelled cells were sorted using flow cytometry and observed with confocal microscopy. A Z-stack was re-sliced horizontally and vertically to obtain the projections of perpendicular views, confirming the intracellular presence of bacteria. This allowed us to observe intracellular *Salmonella* expressing red tag*,* in green labelled cells for all the cells considered. These results confirm the intracellular localization of the different strains and thus validate our protocol designed to identify and quantify the cell types infected by *Salmonella* in selected chick organs. Moreover, they show that *S.* Typhimurium can invade all the cell types studied in this paper (i.e. monocytes–macrophages, B and T lymphocytes, thrombocytes, epithelial and endothelial cells of chicks). Currently, only a few papers have described the cells infected by *Salmonella in vivo* and most of these papers are in mouse models. In these articles, *Salmonella* were found mainly in macrophages and neutrophils from the liver and spleen of mice, but infected B and T lymphocytes were also identified [49–52].

**Figure 2.**
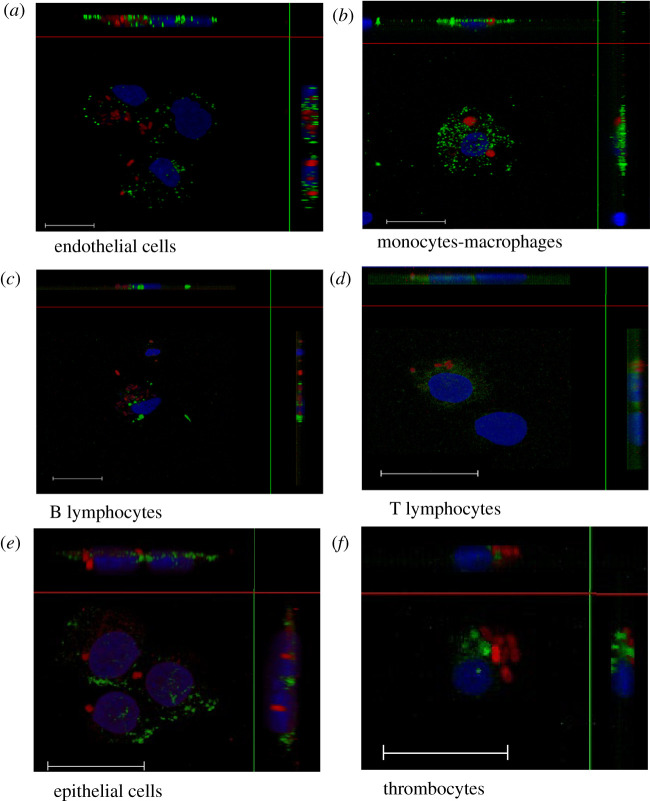
Intracellular localization of *Salmonella* in cells purified from *in vivo* infected organs. Five-day-old chicks were inoculated in the coelomic cavity with around 6 × 10^7^ CFU per chick with *S*. Typhimurium 14028 turboFP650 wild-type strain, Δ*invA*::*kan* mutant strain or the Δ*invA::kan* Δ*pagN::cm* Δ*rck* mutant strain. Two days post infection, animals were sacrificed and the different organs removed. Cells were then isolated from organs, they were sorted using a high-speed cell sorter, MoFlo Astrios EQ and deposited on glass coverslips after cytospin at 200 r.p.m. for 10 min. Next, cells were fixed in formaldehyde. Nucleus staining was performed with Dapi (blue). The bacteria are in red (turboFP650), whereas cells are identified in green due to FITC or Alexa Fluor 488 conjugated antibodies. Cells were observed under a SP8 confocal laser-scanning microscope equipped with a 100× oil immersion objective (Leica). Z-stacks were re-sliced horizontally and vertically to obtain the projections of perpendicular views from three-dimensional images, allowing a view of all bacteria in the cells, using Las AF lite 2.6.3 build 8173 software (Leica). White dashes represent 20 µm. (*a*) Represents endothelial cells from the aortic vessels, infected with the 3Δ strain. Picture size 32.54 × 38.45 µm. (*b*) Represents monocytes–macrophages from the liver, infected with the Δ*invA* strain. Picture size 116.25 × 116.25 µm. (*c*) Represents B lymphocytes, infected with the wild-type strain. Picture size 58.13 × 58.13 µm. (*d*) Represents T lymphocytes, infected with the wild-type strain. Picture size 39.88 × 39.88 µm. (*e*) Represents epithelial cells in the gall bladder, infected with the 3Δ strain. Picture size 37.80 × 37.80 µm. (*f*) Represents thrombocytes in the aortic vessels, infected with the 3Δ strain. Picture size: 116.25 × 116.25 µm.

A more detailed analysis of the confocal images allowed us to observe that in most cases infected cells, whatever the cell type, only harboured one to five bacteria per cell (figure 2), but in a few more bacteria were visualized. This result is consistent with results obtained in the literature on *Salmonella* infected macrophages *in vivo*. Indeed, in many experiments in mice, the majority of liver or spleen infected phagocytes contained relatively few bacteria [49–51,53], but the presence of many bacteria per cell has also been reported [50,53,54]. Our results show that this heterogeneous number of bacteria per cell could be extended to non-phagocytic cells in chicks. However, in mice it seems that the number of bacteria per cell had a moderate impact on the infectious process as host cells that contain high numbers of bacteria have the same probability of undergoing lysis as cells containing only a few bacteria [55]. Both highly and weakly infected cells contribute significantly to the *Salmonella* infection process and not only macrophages [51].

### 3.3. Analysis of the infected cell types in the spleen

In the chicken spleen the distinction between red and white pulp is less marked than in mammals. Red pulp mainly contains erythrocytes, granulocytes, macrophages, scattered T lymphocytes and plasma cells. However, the architecture of avian white pulp differs considerably. Three morphologically distinct areas constitute the spleen. The first consists of peri-arteriolar lymphocyte sheaths, mainly containing T lymphocytes that surround arterioles, which have visible muscular layers. The second involves peri-ellipsoid lymphocyte sheaths (PELS) surrounding capillaries, lacking muscular tissue and lined by cuboidal endothelium and reticulin fibres. The last consists of follicles with germinal centres, surrounded by a capsule of connective tissue. PELS and follicles mainly contain B lymphocytes [56].

**Figure 3.**
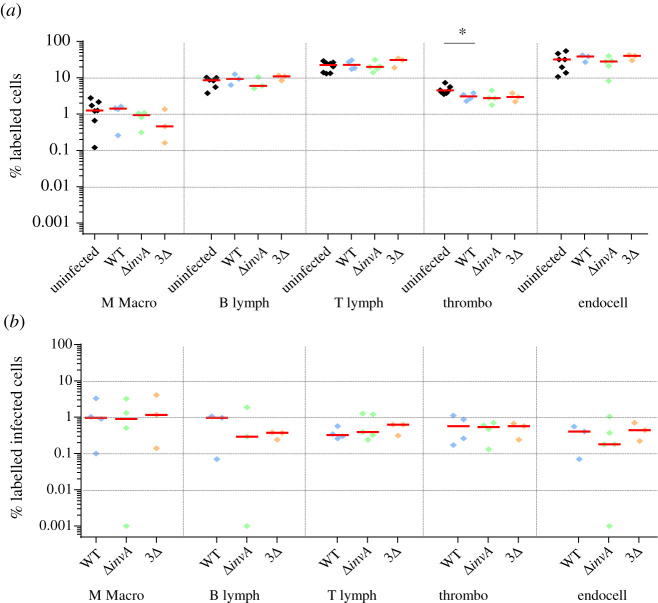
Percentage of identified and *Salmonella* infected cells in spleen 5-day-old chicks were inoculated in the coelomic cavity with around 6 × 10^7^ CFU per chick with *S*. Typhimurium 14028 turboFP650 wild-type strain (WT; blue diamond), Δ*invA*::*kan* mutant strain (Δ*invA*; T3SS-1 defective; green diamond) or the Δ*invA::kan* Δ*pagN::cm* Δ*rck* mutant strain (3Δ; T3SS-1, Rck, PagN defective; yellow diamond). Two days post infection, animals were sacrificed and the different organs removed. Cells from uninfected animals of the same age were used as a control. After labelling with the corresponding antibodies, the percentages of macrophages–monocytes, B and T lymphocytes, thrombocytes and epithelial and endothelial cells were quantified by flow cytometry. The percentage of labelled cells (*a*) and the percentage of labelled infected cells (*b*) are represented. All negative responses were scored at 0.001%. The medians are represented by a red dash. Asymptotic two-sample Fisher–Pitman permutation tests (one-way test) were performed (R software). Significance was *p < 0.05.

In the spleen, the six antibodies used in our study allowed us to detect about 86% of the total cells. Epithelial cell labelling was not necessary, as these cells were not expected to be present. As expected, B lymphocytes (average of 8%), T lymphocytes (average of 22%) and endothelial cells (average of 30%) were identified the most (figure 3*a*). Compared to the non-infected chicks, the percentage of labelled cells was similar in the groups of chicks inoculated with the wild-type bacteria, the single or triple mutant bacteria. The only statistical difference was observed for the percentage of thrombocytes between the uninfected chicks and the chicks infected with the wild-type strain (*p* = 0.049; electronic supplementary material, table S1). The slight decrease in the number of thrombocytes after infection with the wild-type strain, was similar to that observed with the two mutants, but the number of independent experiments was probably not sufficient to obtain a statistical difference between the uninfected group and the chicks inoculated with these *Salmonella* mutants. Similarly, the lower percentage of macrophages observed after infection with the 3Δ mutant strain was not significant. This could be explained either by the absence of cell recruitment in chicks or more probably to the fact that the infected cells were identified only 2 days after bacterial inoculation. The infection rates observed for all the cells identified (lymphocytes, macrophages, thrombocytes and endothelial cells) were between 0.1% and 1%. Monocytes and macrophages were proportionally the most infected cells (about five times more than the other cell types), but endothelial cells, and B and T lymphocyte cells were the most infected cells in the spleen as their absolute number was higher than that of monocytes–macrophages in this organ. No statistical differences were observed between the *Salmonella* strains tested (figure 3*b*). The fact that monocytes–macrophages were identified as being proportionally the most infected cells of the spleen was not surprising. In mice, it is commonly assumed that the systemic spread of *Salmonella* is contingent upon dissemination and survival within macrophages. Indeed, survival in macrophages is essential for virulence [57]. However, contrary to what was assumed, our work clearly demonstrated that other cell types, such as lymphocytes, thrombocytes and endothelial cells of sinusoidal capillaries, could also be infected by *Salmonella* in chicken spleen. As monocytes and macrophages are phagocytic cells, the fact that there was no difference in the percentage of monocyte–macrophage infected cells between the mutants and the wild-type strain was to be expected. By contrast, B and T lymphocytes, thrombocytes and endothelial cells are non-phagocytic cells and thus a difference in the percentage of cells infected by the different strains could have been expected. However, Geddes *et al.* [52] have also described the internalization of *Salmonella* independently of the T3SS-1 in splenic B and T cells of mice. Our work suggests that this observation could be extended to other non-phagocytic cells of other animal species.

### 3.4. Analysis of the infected cell types in the liver

The liver is divided into a right and a left lobe. Each lobe of the liver has approximately 100 000 lobules separated from each other by interlobular septum. These lobules are formed by parenchymal cells (hepatocytes), which represent 80% of the total liver volume, and non-parenchymal cells localized in the sinusoidal wall. These sinusoidal walls are the vascular side of the hepatocytes and they are composed of endothelial cells and macrophages. These macrophages are star-shaped and confined to the liver. Called Kupffer cells, they phagocyte pathogens, cell debris and damaged red and white blood cells [58].

**Figure 4.**
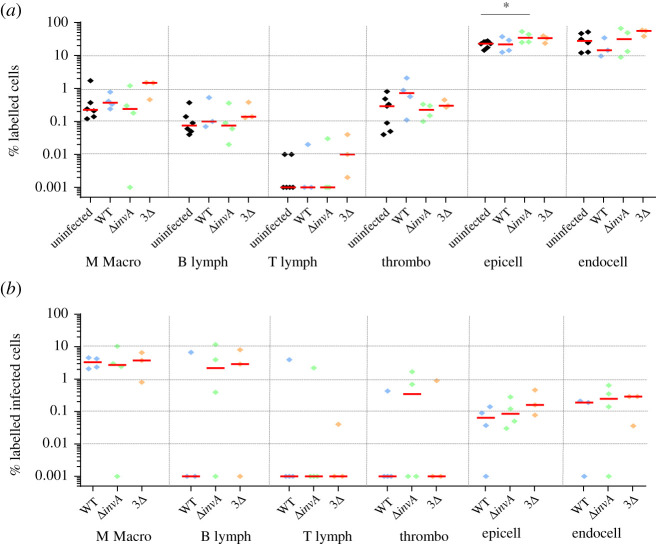
Percentage of identified and *Salmonella* infected cells in liver. Five-day-old chicks were inoculated in the coelomic cavity with around 6 × 10^7^ CFU per chick with *S*. Typhimurium 14028 turboFP650 wild-type strain (WT; blue diamond), Δ*invA*::*kan* mutant strain (Δ*invA*; T3SS-1 defective; green diamond) or the Δ*invA::kan* Δ*pagN::cm* Δ*rck* mutant strain (3Δ; T3SS-1, Rck, PagN defective; yellow diamond). Two days post infection, animals were sacrificed and the different organs removed. Cells from uninfected animals of the same age were used as a control. After labelling with the corresponding antibodies, the percentages of macrophages–monocytes, B and T lymphocytes, thrombocytes and epithelial and endothelial cells were quantified through flow cytometry. The percentage of labelled cells (*a*) and the percentage of labelled infected cells (*b*) are represented. All negative responses were scored at 0.001%. The medians are represented by a red dash. Asymptotic two-sample Fisher–Pitman permutation tests (one-way test) were performed (R software). Significance was *p < 0.05.

In the liver, the six antibodies used allowed us to detect about 76% of the cells and we detected as many epithelial cells (average of 31%) as endothelial cells (average of 33%) (figure 4*a*). As expected, these were the main cell types identified. Few monocytes–macrophages were identified. One hypothesis is that the KUL01 antibody poorly recognizes Kupffer cells [59]. Another explanation could be that their percentage compared to epithelial and endothelial cells is very low in the liver. There were also almost no T lymphocytes. In humans and mice, lymphocytes are present in small quantities at the level of the sinusoids and the space of Disse (perisinusoidal space) and histological investigation does not suggest that there are many immunologically relevant cells present [60]. Liver-resident lymphocytes serve as sentinels and perform immunosurveillance in response to infection and non-infectious insults, and are involved in the maintenance of liver homeostasis [61]. Our low level of T lymphocytes in the liver is most probably related to the fact that our observations were made 2 days after the *Salmonella* inoculation and that our chicks were only 6 days old and therefore immunologically immature. For all cell types in the liver, the percentage of labelled cells was similar, whatever the infected or uninfected status of the animals. Only a statistical difference for the percentage of labelled epithelial cells between the uninfected chicks and those infected with the *invA* mutant was observed (*p* = 0.039; electronic supplementary material, table S1). As in the spleen, we were able to observe similar levels of infected cells between chicks inoculated with the wild-type strain or with the two mutant strains deleted of the known entry factors. Compared to the spleen, there was a greater heterogeneity in the percentages of labelled infected cells (figure 4*b*). In particular, the percentages of infected monocytes–macrophages and B lymphocytes were around 3%, while those of infected epithelial and endothelial cells were 0.10% and 0.24%, respectively. Nevertheless, as the two latter cell types are more frequent in the liver than monocytes–macrophages and B lymphocytes (figure 4*a*), endothelial cells of sinusoidal capillaries and epithelial cells represent a large proportion of the infected cells in the liver. One of the roles of liver monocytes–macrophages is the phagocytosis of pathogens and thus it is not surprising that they were found infected, with no differences between any of the strains inoculated. By contrast, thrombocytes were very weakly infected here.

Overall, these results strengthen those observed in the spleen, showing that at least four different cell types (i.e. monocytes–macrophages, B lymphocytes, endothelial and epithelial cells) were infected by *Salmonella* in the liver of chicks.

### 3.5. Analysis of the infected cell types in the aortic vessels

The term ‘aortic vessels' in our paper corresponds to the aortic arch and the brachiocephalic trunk. By contrast to mammals, two brachiocephalic trunks arise from the arch of the aorta and give rise to the common carotid and subclavian arteries in birds [62]. For this organ, only 55% of the cells were identified through flow cytometric analysis. This low percentage of identification was mainly related to the presence of smooth muscle fibres in vessels for which no antibodies exist for the chicken. The adventitia, which is the outer layer of the arterial wall, is made up of connective tissue and elastic fibres. It contains capillary vessels vascularizing the arterial wall as well as nerve fibres of the sympathetic and parasympathetic autonomic system. According to the size of the arteries, the media, which is the middle layer of the arterial wall, is made up of collagen, elastin or smooth muscle fibres allowing vasoconstriction. The intima, the inner layer of the arterial wall separated from the media by the internal elastic limiter, consists of vascular endothelium (cell monolayer) resting on a layer of connective tissue [63].

**Figure 5.**
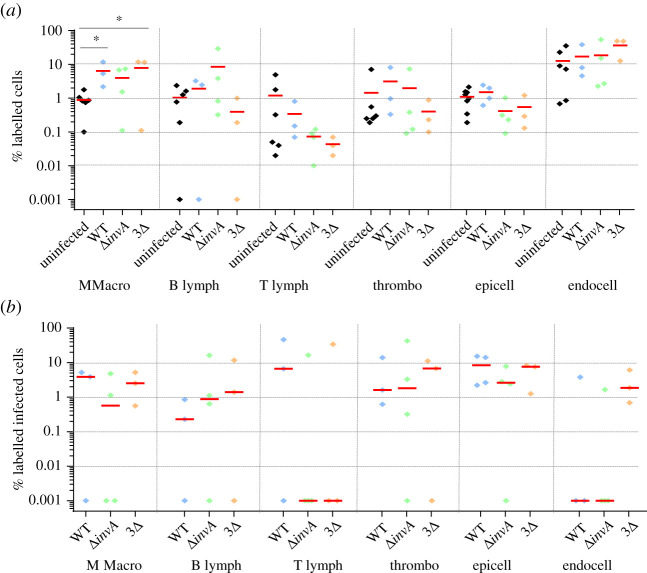
Percentage of identified and *Salmonella* infected cells in aortic vessels. Five-day-old chicks were inoculated in the coelomic cavity with around 6.10^7^ CFU per chick with *S*. Typhimurium 14028 turboFP650 wild-type strain (WT; blue diamond), Δ*invA*::*kan* mutant strain (Δ*invA*; T3SS-1 defective; green diamond) or the Δ*invA::kan* Δ*pagN::cm* Δ*rck* mutant strain (3Δ; T3SS-1, Rck, PagN defective; yellow diamond). Two days post infection, animals were sacrificed and the different organs removed. Cells from uninfected animals of the same age were used as a control. After labelling with the corresponding antibodies, the percentages of macrophages–monocytes, B and T lymphocytes, thrombocytes and epithelial and endothelial cells were quantified by flow cytometry. The percentage of labelled cells (*a*) and the percentage of labelled infected cells (*b*) are represented. All negative responses were scored at 0.001%. The medians are represented by a red dash. Asymptotic two-sample Fisher–Pitman permutation tests (one-way test) were performed (R software). Significance was *p < 0.05.

The percentages of cells labelled in aortic vessels according to the chick group illustrated in figure 5*a* varied more than for the previous two organs, probably due to the treatment of the aortic vessel with enzymes, which made extraction more difficult. Monocytes–macrophages and endothelial cells (of continuous capillaries) were the most representative type of cells labelled, but all cell types were identified (figure 5*a*). In this organ, we observed a difference between the percentages of monocytes–macrophages according to the uninfected or infected status of the animals. Contrary to the spleen and the liver, the percentage of labelled monocytes–macrophages showed statistically significant differences between the uninfected chicks and those infected with the wild-type strain, on the one hand (*p* = 0.035; electronic supplementary material, table S1), and those infected with the 3Δ mutant, on the other hand (*p* = 0.043; electronic supplementary material, table S1). Despite a high percentage of labelled endothelial cells, few if any were infected. By contrast, all the other cell types were infected and to a greater extent than in the spleen and liver (figure 5*b*). Indeed, compared to the spleen and liver, the median percentage of each infected cell type in the aortic vessels, except endothelial cells, varied from 1% to 10% versus 0.1–1% in the spleen or 0–3% in the liver. Surprisingly, in some chicks more than 10% of lymphocytes, thrombocytes and epithelial cells were infected.

These results clearly show that, like in the spleen and liver, numerous cell types are infected in vessels.

### 3.6. Analysis of the infected cell types in the gall bladder

The avian gall bladder is attached to the right liver lobe. Histologically, it is composed of three tunicae. The first, the *tunica mucosa* is mainly lined with non-ciliated simple columnar epithelium and consists of a layer of connective tissue with elastic and muscle fibres. The second, the *tunica muscularis* consists of smooth muscle fibres and abundant intervening connective tissue. The third, the *tunica serosa*, consists of coarse collagen fibre and elastic fibres. All epithelial cells are basally located and contain an oval nucleus. Bile is synthesized in the hepatocytes and secreted into bile canaliculi located on the lateral surfaces of adjoining liver cells [64]. Relatively little is known about biliary secretion in birds due to the complex anatomy in which bile enters the intestine via both hepato-enteric and cystico-enteric ducts. In ruminants, pigs and poultry, there is a relatively continuous secretion of bile into the intestine [58].

**Figure 6.**
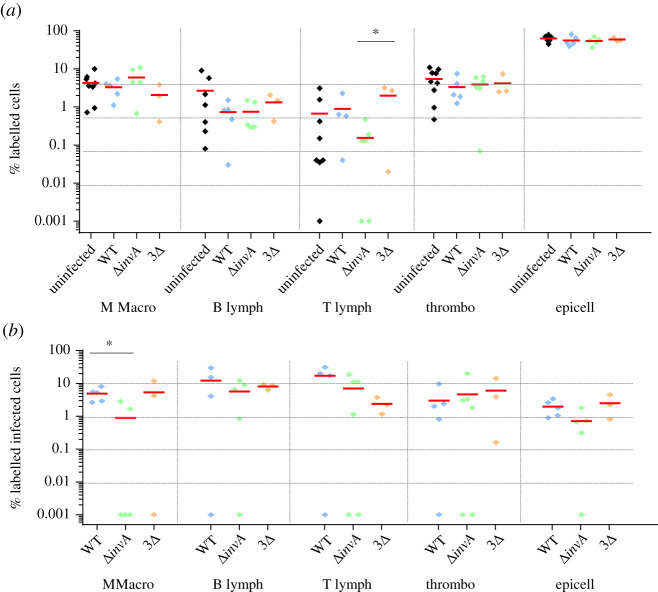
Percentage of identified and *Salmonella* infected cells in gall bladder. Five-day-old chicks were inoculated in the coelomic cavity with around 6.10^7^ CFU per chick with *S*. Typhimurium 14028 turboFP650 wild-type strain (WT; blue diamond), Δ*invA*::*kan* mutant strain (Δ*invA*; T3SS-1 defective; green diamond) or the Δ*invA::kan* Δ*pagN::cm* Δ*rck* mutant strain (3Δ; T3SS-1, Rck, PagN defective; yellow diamond). Two days post infection, animals were sacrificed and the different organs removed. Cells from uninfected animals of the same age were used as a control. After labelling with the corresponding antibodies, the percentages of macrophages–monocytes, B and T lymphocytes, thrombocytes and epithelial and endothelial cells were quantified by flow cytometry. The percentage of labelled cells (*a*) and the percentage of labelled infected cells (*b*) are represented. All negative responses were scored at 0.001%. The medians are represented by a red dash. Asymptotic two-sample Fisher–Pitman permutation tests (one-way test) were performed (R software). Significance was *p < 0.05.

About 75% of cells were identified with the available antibodies. Numerous ‘unidentified cells' would most probably correspond to fibroblasts. All the cell types studied were identified. The epithelial cells represented about 60% of the identified cells and the percentages of thrombocytes and monocytes–macrophages were between 1% and 10% (figure 6*a*). B and T lymphocytes were present to a lesser extent. As for the aortic vessels, the variability between animals was considerable, certainly due to the breakdown of organs with different enzymes, which made extraction less reproducible. As in the other organs (except for the percentages of monocytes–macrophages in the aortic vessels), there were no differences in the percentages of the labelled cells between uninfected and infected chicks. When comparing the inoculated chicks, only one statistically significant difference was observed regarding the percentage of T lymphocytes between the chicks inoculated with the single or the triple mutant (*p* = 0.041; electronic supplementary material, table S1). Immune cells were highly infected. For example, about 10% of the B and T lymphocytes were infected by the different strains. Moreover, the gall bladder B and T lymphocytes were found to be infected more than in the other organs. The other identified cells were infected between 1% and 10% (figure 6*b*). In all cases except one, no statistical differences were observed between the chicks inoculated with the wild-type or the mutant strains. The only significant difference was observed for the infected monocytes–macrophages between the chicks infected with the wild-type strain and those infected with the *invA* mutant strain (*p* = 0.020; electronic supplementary material, table S1). This could be due to the fact that only two Δ*invA-*inoculated animals had infected monocytes–macrophages, while the percentages of monocytes–macrophages were similar for the five animals tested. Another interesting point is that the analyses of the infected areas (labelled + unlabelled) highlighted the high cell invasion rates of the gall bladder (table 1). Combining all experiments, after 2 days of infection, the median percentages of all infected cells (labelled and unlabelled) in the gall bladder were 2.23%, 1% and 3.65% depending on the strain inoculated, whereas in the spleen, for example, they were only 0.35%, 0.19% and 0.31%.

**Table 1.** Percentages of infected cells (labelled and unlabelled) according to the organ.

	spleen	liver	aortic vessels	gall bladder
	median (Q1; Q3)	median (Q1; Q3)	median (Q1; Q3)	median (Q1; Q3)
WT	0.35 (0.19; 0.53)	0.07 (0.06; 0.10)	0.64 (0.42; 2.49)	2.23 (1.54; 2.82)
Δ*invA*	0.19 (0.14; 0.55)	0.06 (0.04; 0.16)	0.21 (0.12; 1.46)	1.00 (0.52; 2.71)
3Δ	0.31 (0.26; 0.38)	0.06 (0.04; 0.16)	2.18 (1.18; 2.54)	3.65 (2.39; 4.62)

**Figure 7.**
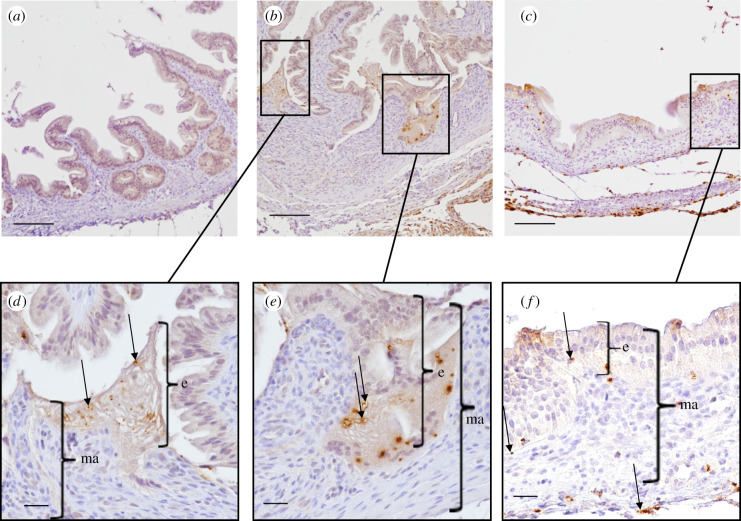
Immunohistochemistry of chick gall bladder infected with *S. Typhimurium* wild-type stain or with a mutant deleted of the three known invasion factors 5-day-old chicks were inoculated in the coelomic cavity with around 6 × 10^7^ CFU per chick with *S*. Typhimurium 14028 turboFP650 wild-type strain (WT) or the Δ*invA::kan* Δ*pagN::cm* Δ*rck* mutant strain (3Δ). Two days post infection, animals were sacrificed. Gall bladders were removed and fixed in 4% buffered paraformaldehyde at 4°C for 24 h. Tissues were processed using routine methods, paraffin embedded, cut in sections (thickness, 5 µm) and stained with diaminobenzidine for IHC with HRP detection. The primary antibody was a rabbit anti-*Salmonella* O:4,5 lipopolysaccharide marker. Tissues were examined and photographed with a light microscope Eclipse 80i, Nikon with DXM 1200C digital camera (Nikon Instruments, Europe, Amsterdam, The Netherlands) and NIS-Elements D Microscope Imaging Software. Tissues were counterstained in blue with Harris's haematoxylin and *Salmonella* were stained in brown with HRP detection. Representative pictures are presented. Bacteria are seen (→) within the epithelium (e) and the mucosa (ma). Sections of a gall bladder of (*a*) an uninfected chick, (*b*,*d*,*e*) a chick infected by the wild-type strain and (*c*,*f*) a chick infected by the 3Δ mutant are represented. Scale bar, 100 μm in (*a*–*c*) and 20 μm in (*d*–*f*).

As this organ has never previously been described as a site of *Salmonella* colonization in chicks and as it is described as an organ that is important for *Salmonella* persistence in mice and humans [65,66], we decided to observe the infected tissues using immunohistochemistry. Microscopic analysis shows that bacteria were located in the *mucosa* of the gall bladder including in the epithelium but also in the *lamina propria*, whatever the strain inoculated. Interestingly, when high numbers of *Salmonella* were detected the epithelium was damaged, while the structure of the gall bladder was well conserved when the tissue was only infected by a few bacteria (figure 7).

The gall bladder is thus colonized by *Salmonella* after chicks are inoculated via an intracoelomic route. These results show that oral inoculation of *Salmonella* is not necessary for gall bladder infection. The bacteria reached the gall bladder through the vasculature or the ducts that emanate from the liver. Menendez *et al*. [67] obtained similar results in a mouse infection model. Indeed, they demonstrated that gallbladder colonization was not the result of *Salmonella* ascending directly from the gastrointestinal tract and their histological analyses supported the idea that bacteria were discharged from the liver into the gall bladder *via* the bile. Concerning the infected cells in the chick gall bladder, monocytes–macrophages, B and T lymphocytes, thrombocytes and epithelial cells of this organ were all infected at a relatively high level compared to the other organs and epithelial cells were the most infected cell type. Menendez *et al.* [67] also observed in their mouse model that *Salmonella* localized preferentially within epithelial cells of the gallbladder. However, bacteria were rarely seen within the *lamina propria*. Our observations of some *S*. Typhimurium in the *mucosa* and *submucosa* and the identification of infected monocytes–macrophages, B and T lymphocytes and thrombocytes demonstrate that epithelial cells are not the only cells infected in the gall bladder of chicks. Whether this result is restricted to chicks remains to be determined. Our results on our mutant strains also differ from those of Menendez *et al*. [67]. Indeed, our mutants were shown to infect similar cells to the wild-type, while Menendez *et al.* did not observe their *inv*A mutant in the epithelial cells of the murine gall bladder, in contrast to their wild-type strain, suggesting that the T3SS-1 is required for *Salmonella* colonization of the gall bladder in mice but not in chicks.

The gall bladder is known to be an organ in which *S*. Typhi persist during chronic infections in humans, after forming a biofilm on gallstones [40,66,68]. Models of chronic infection in mice have also been studied [65,67,69]. In guinea pigs, although asymptomatic, *Salmonella* could be recovered in the gall bladder for up to five months post infection [70]. However, in chicken it is not known whether this organ could be relevant for the persistence of *Salmonella*.

### 3.7. *Salmonella* Typhimurium is able to persist in the gall bladder independently of the T3SS1, Rck and PagN

**Figure 8.**
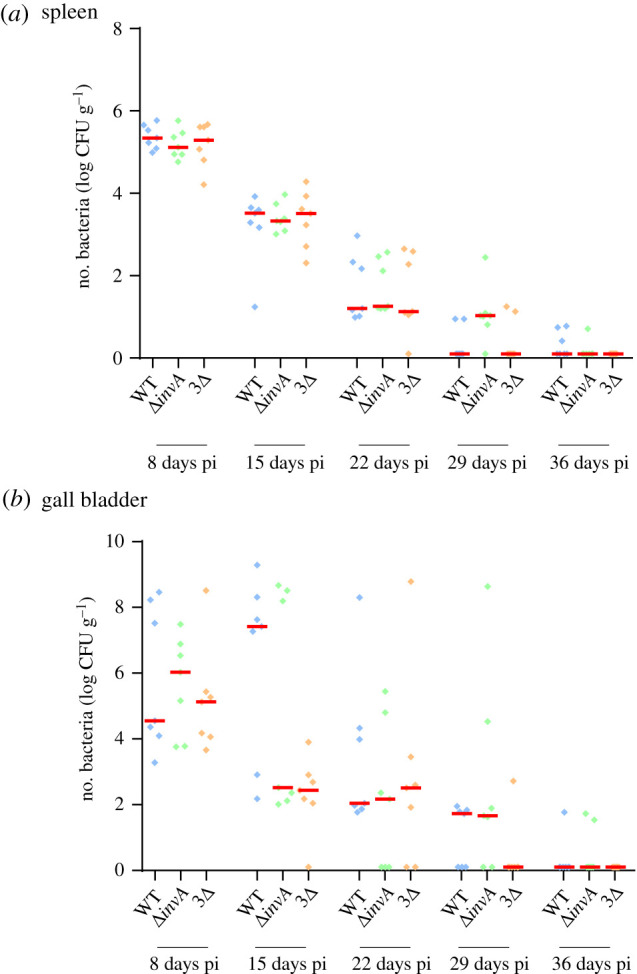
Persistence of *S.* Typhimurium in the spleen and in the gall bladder after intracoelomic inoculation. Five-day-old chicks were inoculated in the coelomic cavity with around 3 × 10^7^ CFU per chick with *S*. Typhimurium 14028 turboFP650 wild-type (WT; blue diamond), Δ*invA*::*kan* mutant strain (Δ*invA*; T3SS-1 defective; green diamond) or the Δ*invA::kan* Δ*pagN::cm* Δ*rck* mutant strain (3Δ; T3SS-1, Rck, PagN defective; yellow diamond). Each week, seven animals were sacrificed and their spleens and gall bladders removed. The kinetics of spleen (*a*) and gall bladder (*b*) colonization were monitored each week for a period of 36 days. Results are expressed as number of bacteria (log CFU per gram of organ). The medians are represented by a red dash. A Kruskal–Wallis test was conducted, followed by a Dunn's multiple comparisons test (GraphPad Software). Significance was *p < 0.05.

As the previous results demonstrated that bacterial concentrations in the gall bladder were significant (figure 1) and that *Salmonella* was able to infect several cell types in this organ, we verified whether this organ could be infected over the long term. To determine persistence, we infected chicks and monitored their colonization rate in the spleen and gall bladder for 36 days with slaughtering every 8 days. Bacteria were detected throughout the kinetics. No statistical differences of colonization could be observed in the spleen (figure 8*a*) or in the gall bladder (figure 8*b*), whatever the strain inoculated and the week of analysis. This work demonstrates for the first time that *Salmonella* Typhimurium could invade the gall bladder of chicks at levels and durations similar to those observed in the spleen and thus, can be considered as a site of colonization in addition to the spleen and the liver in chicks. In mice, during chronic infection, mimicking human *S.* Typhi infection, the spleen and the gall bladder are considered as organs of persistence and gall bladder colonization presumably leads to re-infection of the intestine through bile secretion [39,54,65]. Our results also demonstrate that for the gall bladder to be infected an oral route of infection is not necessary, as demonstrated by Menendez *et al*. [67] in a mouse model. The role of colonization of this organ needs to be analysed further, particularly regarding intestinal colonization and persistence of *Salmonella*.

## 4. Conclusion and opening up

This work demonstrates for the first time, that *S.* Typhimurium can invade *in vivo* a large array of phagocytic and non-phagocytic cells of different organs and vessels in chicks. These cells are immune cells but also epithelial and endothelial cells as previously demonstrated *in vitro* with cell lines [31]. Moreover, numerous unidentified cells were infected. This is explained by the lack of antibodies in chicken to identify among others, dendritic cells, fibroblasts and heterophils, which are also important for the spread of bacteria [54,71,72]. Therefore, new antibodies need to be developed for further studies in chicks. Nevertheless, our results show a great difference between mice and chicks. In mice, phagocytic cells and especially macrophages are the main cells in which *Salmonella* replicate in the liver and spleen [49–51,54]. In chicks, cell tropism in these organs, and also in the gall bladder and vessels, is more diverse with *Salmonella* being found intracellularly not only in monocytes–macrophages but also in lymphocytes, and endothelial and epithelial cells. Interestingly, endothelial cells from sinusoid capillaries (spleen and liver) were targeted by *Salmonella*, while endothelial cell invasion into continuous capillaries (vessels) was less clear and remains to be confirmed. Whether macrophages are or not the privileged localization of *Salmonella* in organs other than the liver and spleen in mice remains to be determined. Chicken cells infected in the gastrointestinal tract by *Salmonella* were not studied in this paper despite the relevance of this organ in *Salmonella* infection. This was due to serious experimental difficulties encountered including the auto-fluorescence of intestinal cells. Work is currently in progress to overcome these problems.

Even if *Salmonella* are able to invade numerous cells, specificity exists depending on the organ. Indeed, for example, during *Salmonella* infection in chicks, epithelial cells appear more sensitive in the gall bladder than in the liver. Similarly, endothelial cells appear more sensitive in the spleen than in the aortic vessels.

Surprisingly, the two mutant strains used in this study (i.e. a T3SS-1 mutant strain and a mutant strain defective for the three currently known invasion factors) were able to invade the same host cells as the wild-type strain. The fact that the triple mutant strain entered numerous host cells, *in vivo*, confirms our previous results suggesting the existence of unknown invasion factors [31]. However, we cannot conclude that the T3SS-1, PagN and Rck are not required for the invasion of chicken cells as a redundant role of the different invasion factors may occur. These two hypotheses are reinforced by our results of chicken infection demonstrating that chicks can be colonized at a higher level by the two mutant strains than by their wild-type parent after bacterial inoculation in the coelomic cavity. The absence of T3SS-1 requirement for chicken colonization has already been observed [18–20] and this fact can now be extended to the PagN and Rck invasins. However, in order to demonstrate whether or not these entry factors are redundant, further studies are necessary in chicks and other animals. Altogether, these results are important in understanding the mechanisms of *Salmonella* pathogenesis, as previous work has described that both bacterial behaviour and host response are different [28] depending on the entry mechanism, and thus this opens up new avenues of research. It raises the question as to whether possible unknown bacterial factors are required for cell invasion of systemic sites in chicken and, if this is the case, whether *in vivo* certain cell types are infected by a particular entry mechanism. It also questions whether the known invasion factors in chicks can replace each other. Finally, the fact that cells can be infected through multiple pathways in an organ suggests that their response could be multiple. Further studies involving Tnseq-mutant library screenings and single cell approaches would help to address these questions.

In conclusion, this paper demonstrates that *S.* Typhimurium infects numerous phagocytic and non-phagocytic cells in the spleen, liver and vessels of chicks. Cell invasion of the chick gall bladder was also observed for the first time. Monocytes–macrophages, B and T lymphocytes, thrombocytes and epithelial cells of this organ were all infected and at higher cell invasion rates than the other organs. Moreover, invasion of these cells occurs even in the absence of the three entry factors already identified whatever the organ. In line with these results, *S.* Typhimurium was shown to persist in the gall bladder of chicks independently of the three known invasion factors.

## Data accessibility

All the data of flow cytometry, immuno-histochemistry and confocal microscopy have been deposited in the data repository Data INRAE at https://doi.org/10.15454/OEG7WH.

The data are provided in the electronic supplementary material [73].
